# Metabolic and Antioxidant Modulation by *Artemisia indica* Willd. Aqueous Extract in Glucose and Cholesterol Dysregulation

**DOI:** 10.3390/ijms27010297

**Published:** 2025-12-27

**Authors:** Chiao-Yun Tseng, Yueching Wong, Charng-Cherng Chyau, Yu-Hsuan Liang, Hui-Hsuan Lin, Jing-Hsien Chen

**Affiliations:** 1 Department of Nutrition, Chung Shan Medical University, Taichung City 40201, Taiwan; 1146002@live.csmu.edu.tw (C.-Y.T.); wyc@csmu.edu.tw (Y.W.); 1346003@live.csmu.edu.tw (Y.-H.L.); 2 Research Institute of Biotechnology, Hungkuang University, No. 1018, Sec. 6, Taiwan Boulevard, Shalu District, Taichung City 43302, Taiwan; ccchyau@hk.edu.tw; 3 Department of Medical Laboratory and Biotechnology, Chung Shan Medical University, Taichung City 40201, Taiwan; linhh@csmu.edu.tw; 4 Department of Medical Research, Chung Shan Medical University Hospital, Taichung City 40201, Taiwan

**Keywords:** *Artemisia indica* Willd., glucose dysregulation, cholesterol dysregulation, polyol pathway, antioxidant activity

## Abstract

*Artemisia indica* Willd., a traditional medicinal and dietary herb, has been widely recognized for its diverse bioactivities. This study aimed to evaluate the effects of *Artemisia indica* Willd. aqueous extract (AAE) on dysglycemia and dyslipidemia. HPLC–ESI–MS/MS analysis identified 4,5-dicaffeoylquinic acid as the major active constituent of AAE. BALB/cByJNarl mice subjected to a high-fat diet (HFD) and streptozotocin (STZ) injection were supplemented with AAE for 6 weeks. To elucidate the underlying mechanisms, we examined multiple metabolic pathways, including oxidative stress, lipid metabolism, and the polyol pathway. AAE administration attenuated fasting blood glucose and reduced fructosamine levels and also ameliorated protein kinase C α (PKC-α) and nuclear factor kappa B (NF-κB) expression. Histopathological evaluation showed that AAE reduced lipid accumulation by modulating sterol regulatory element-binding protein 1 (SREBP-1) and fatty acid synthase (FAS) expression. Additionally, AAE inhibited polyol pathway activation and restored antioxidant enzyme activities. Collectively, these findings indicate that AAE modulates glucose and cholesterol metabolism, attenuates oxidative stress, and improves metabolic homeostasis, supporting its potential as a natural herbal therapeutic agent.

## 1. Introduction

Glycemia resulting from β-cell dysfunction is a hallmark of metabolic dysregulation and disrupts glucose, lipid, and protein homeostasis, increasing the risk of metabolic and renal complications [1]. High-fat diet (HFD) feeding induces insulin resistance, and when combined with streptozotocin (STZ), it further impairs β-cell function, modeling human metabolic disturbances [2]. Excess glucose activates the polyol pathway, leading to aldose reductase-mediated fructose production and oxidative stress [3]. Elevated reactive oxygen species (ROS) activate protein kinase C (PKC) and nuclear factor kappa B (NF-κB) while reducing antioxidant defenses. Typical dyslipidemia refers to abnormal serum levels of cholesterol or triglycerides and further aggravates oxidative stress through sterol regulatory element-binding protein (SREBP)-driven lipid biosynthesis [4]. Despite available treatments, managing glucose and cholesterol dysregulation remains challenging due to limited efficacy and treatment-associated adverse effects.

*Artemisia indica* Willd., a perennial herb from Asia including China, India, Taiwan, and Japan, is traditionally cooked with barley or added to rice to enhance flavor and color and also used by local people to alleviate chronic fever, as an anthelmintic, and for hepatobiliary ailments. In Japan, *Artemisia indica* Willd. is a part of the traditional diet; it contributes to the local food culture alongside other medicinal plants [5]. In previous studies, *Artemisia indica* Willd. extract (AAE) exhibited a wide variety of biological activities, including antifungal and anti-inflammatory activity [6,7,8]. The therapeutic effects of medicinal plant prescriptions and bioactivity of Chinese herbs have gained considerable recognition in the development of new drugs. Medicinal plants have long been recognized for their therapeutic potential, offering advantages such as lower toxicity and fewer side effects compared with conventional pharmacological treatments. Plant-derived agents, enriched with bioactive constituents such as flavonoids, terpenoids, and phenolic compounds, have shown considerable potential in regulating metabolic disturbances and suppressing oxidative stress [9]. Bioactive compounds from Chinese medicinal herbs have gained attention as potential agents capable of targeting multiple metabolic pathways [10]. Consequently, interventions that enhance antioxidant defenses while improving glucose and cholesterol regulation may provide metabolic benefits. This study examines whether the aqueous extract of *Artemisia indica* Willd. (AAE) modulates oxidative stress and pathways involved in glucose and cholesterol dysregulation.

## 2. Results

### 2.1. Identification and Quantification of Phytochemical Constituents of Artemisia indica Willd. Aqueous Extract (AAE) by HPLC-ESI-MS/MS

**Figure 1:**
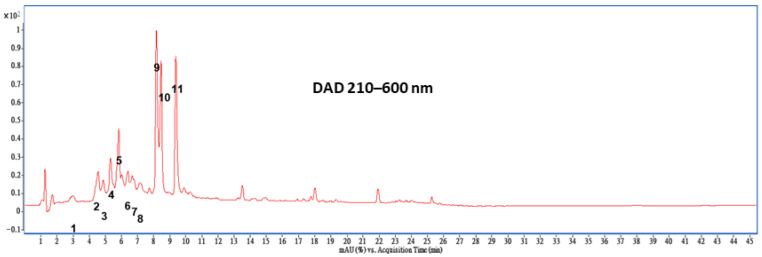
Identification and quantification of phytochemical constituents of *Artemisia indica* Willd. aqueous extract (AAE) by HPLC-ESI-MS/MS.

To analyze the composition of *Artemisia indica* Willd. aqueous extract (AAE), the HPLC-ESI-MS/MS method was utilized. The HPLC-DAD chromatographic profiles of AAE samples are presented in Figure 1, with the corresponding chromatograms and identified compounds listed in Figure 1 and Table 1. Among the compounds identified in AAE, 4,5-dicaffeoylquinic acid (212.6 mg/100 g dry weight (DW)), 3,4-dicaffeoylquinic acid (179.3 mg/100 g DW), and 3,5-dicaffeoylquinic acid (137.4 mg/100 g DW) were found to be the most abundant in AAE.

### 2.2. Effects of AAE on Glycemic Control and Renal Function in HFD/STZ-Induced Mice

**Figure 2:**
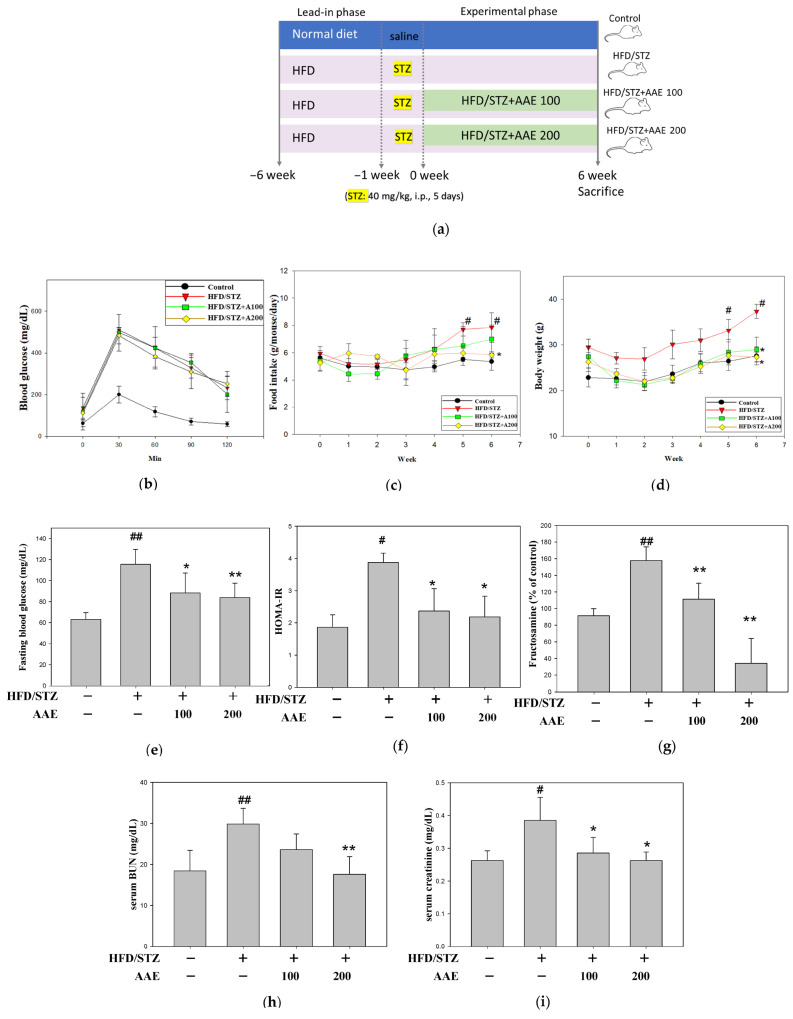

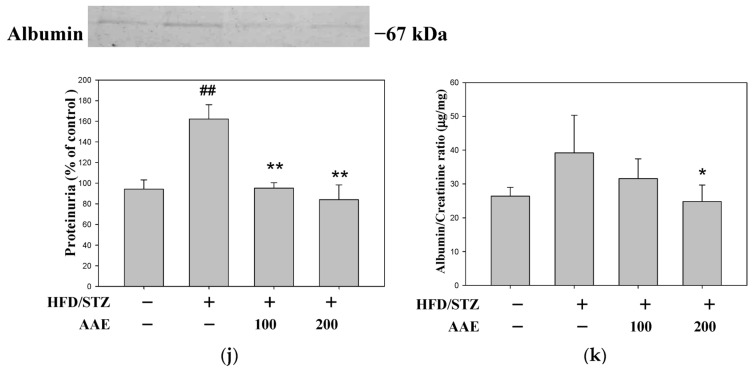
Effects of AAE on glycemic control and renal function in HFD/STZ-induced mice. (**a**) Experimental design: BALB/cByJNarl mice were fed a high-fat diet (HFD) for 6 weeks and subsequently injected intraperitoneally with streptozotocin (STZ, 40 mg/kg, for five consecutive days). Mice were then treated with AAE (100 or 200 mg/kg, *w*/*w* in feed, administered daily) for 6 weeks. After sacrifice, serum and urine samples were collected for renal function assessment. (**b**) Oral glucose tolerance test (OGTT) before treatment with AAE. (**c**) Food intake. (**d**) Body weight. (**e**) Fasting blood glucose. (**f**) Homeostatic model assessment of insulin resistance (HOMA-IR). (**g**) Serum fructosamine. (**h**) Serum blood urea nitrogen (BUN). (**i**) Serum creatinine. (**j**) Urinary protein excretion evaluated by SDS-PAGE. (**k**) Urine albumin-to-creatinine ratio (UACR). Data were collected per group and are presented as the mean ± SD (*n* = 5), derived from five independent biological replicates. ^#^ *p* < 0.05, ^##^ *p* < 0.01 versus the control group; * *p* < 0.05, ** *p* < 0.01 versus the model group. AAE: *Artemisia indica* Willd. aqueous extract; BUN: blood urea nitrogen; HFD: high-fat diet; HOMA-IR: homeostatic model assessment of insulin resistance; i.p.: intraperitoneal injection; STZ: streptozotocin; UACR: urine albumin-to-creatinine ratio.

The experimental design is illustrated in Figure 2a. As shown in Figure 2b, an oral glucose tolerance test (OGTT) was conducted in all groups prior to AAE treatment to confirm successful establishment of the metabolic model. The HFD/STZ group showed a significant increase in both food intake and body weight (Figure 2c,d). Fasting blood glucose was measured immediately after sacrifice. AAE treatment reduced fasting blood glucose (Figure 2e) and improved insulin resistance, as demonstrated by a decrease in the homeostatic model assessment of insulin resistance (HOMA-IR) (Figure 2f), while also lowering serum fructosamine (Figure 2g), collectively indicating improved glycemic control. In addition, AAE administration markedly decreased serum blood urea nitrogen (BUN) and creatinine, suggesting an improvement in renal function (Figure 2h,i). Furthermore, urinary protein analysis by SDS-PAGE with Coomassie blue staining revealed a reduction in proteinuria in the AAE-treated groups (Figure 2j). Furthermore, quantification of the urine albumin-to-creatinine ratio (UACR) showed an increasing trend, whereas a high dose of AAE treatment produced a reducing tendency (Figure 2k).

### 2.3. AAE Improved Renal Histological Alterations and Ameliorated PKC-α/NF-κB Expression in HFD/STZ Mice

**Figure 3:**
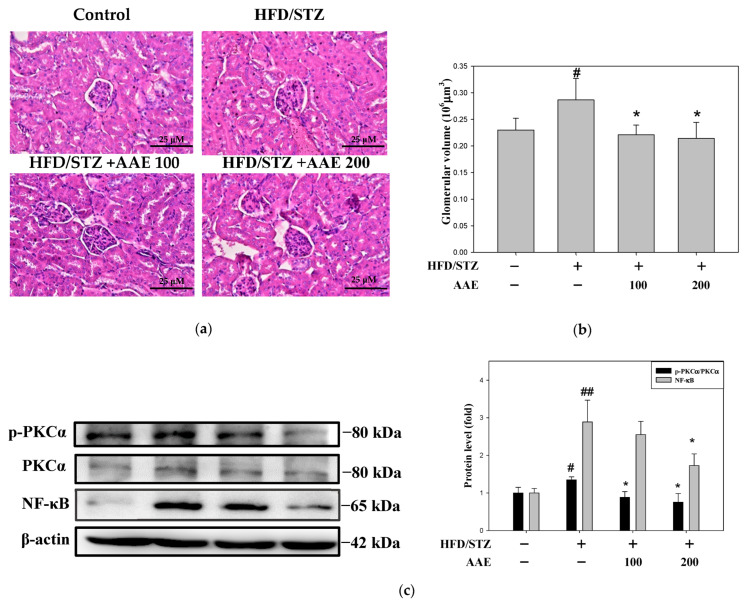
AAE improved renal histological alterations and ameliorated PKC-α/NF-κB expression in HFD/STZ mice. (**a**) The pathological changes were examined through hematoxylin and eosin staining (400× magnification). (**b**) The quantification of glomerular volume. (**c**) Western blot analysis was performed to determine the protein levels of p-PKCα, PKCα, and NF-κB, with β-actin used as a loading control. Data were collected per group and are presented as the mean ± SD (*n* = 5), derived from five independent biological replicates. ^#^ *p* < 0.05, ^##^ *p* < 0.01 versus the control group; * *p* < 0.05 versus the model group. AAE: *Artemisia indica* Willd. aqueous extract; HFD: high-fat diet; NF-κB: nuclear factor kappa B; p-PKCα: phosphorylated protein kinase C; PKCα: protein kinase C α; STZ: streptozotocin.

Representative photomicrographs of kidney sections stained with hematoxylin and eosin demonstrated that AAE treatment ameliorated glomerular enlargement (Figure 3a). Quantitative analysis revealed that glomerular volume was increased in the HFD/STZ group compared with the control group, whereas AAE treatment restored glomerular volume in mice (Figure 3b). The phosphorylated protein kinase C (p-PKC-α)/protein kinase C (PKC-α) expression ratio was elevated in the HFD/STZ group, whereas AAE administration was associated with a dose-dependent attenuation of this increase. In addition, HFD/STZ treatment was accompanied by an elevation in the p-PKC-α/PKC-α level together with a concomitant increase in nuclear factor-κB (NF-κB) expression. High-dose AAE treatment was observed to partially modulate NF-κB expression (Figure 3c).

### 2.4. AAE Decreased Lipid Accumulation by Modulating SREBP-1 and FAS Expression in HFD/STZ Mice

**Figure 4:**
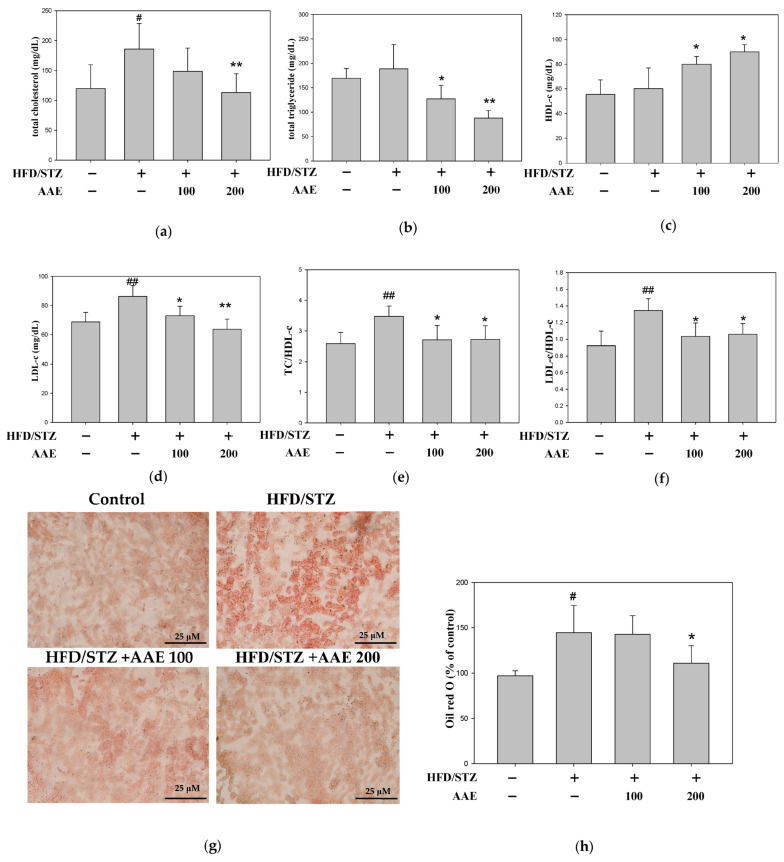

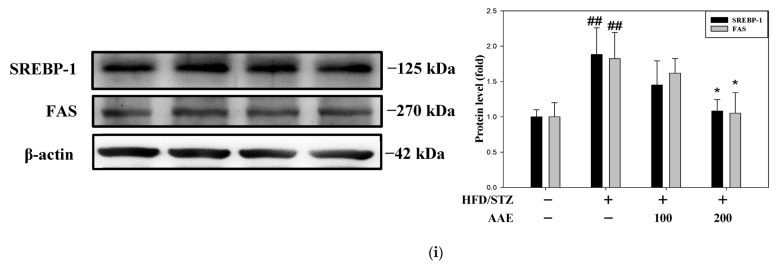
AAE decreased lipid accumulation by modulating SREBP-1 and FAS expression in HFD/STZ mice. (**a**) Total cholesterol (TC). (**b**) Total triglycerides (TGs). (**c**) High-density lipoprotein cholesterol (HDL-c). (**d**) Low-density lipoprotein cholesterol (LDL-c). (**e**) The ratio of TC/HDL-c. (**f**) The ratio of LDL-c/HDL-c. (**g**) The pathological changes examined through Oil Red O illustrate lipid accumulation (400× magnification). (**h**) Oil Red O-positive area. (**i**) Western blot analysis was performed to determine the protein levels of SREBP-1 and FAS, with β-actin used as a loading control. Data were collected per group and are presented as the mean ± SD (*n* = 5), derived from five independent biological replicates. ^#^ *p* < 0.05,^##^ *p* < 0.01 versus the control group; * *p* < 0.05, ** *p* < 0.01 versus the model group. AAE: *Artemisia indica* Willd. aqueous extract; FAS: fatty acid synthase; HFD: high-fat diet; SREBP-1: sterol regulatory element-binding protein 1; STZ: streptozotocin.

AAE treatment reduced serum lipid biomarkers, including total cholesterol (TC) (Figure 4a), total triglycerides (TGs) (Figure 4b), and low-density lipoprotein cholesterol (LDL-c) (Figure 4d), as well as the TC/HDL-c (Figure 4e) and LDL/HDL (Figure 4f) ratios, while concomitantly increasing high-density lipoprotein cholesterol (HDL-c) (Figure 4c). Representative Oil Red O-stained images showed abundant lipid droplets (red signals) in the renal tissue of HFD/STZ mice, indicating lipid accumulation (Figure 4g). Quantitative analysis revealed that AAE treatment markedly decreased the lipid-droplet-positive area (Figure 4h). Furthermore, Western blot analysis showed that the elevated expression of sterol regulatory element-binding protein 1 (SREBP-1) and fatty acid synthase (FAS) in the kidneys of HFD/STZ mice appeared to be attenuated by AAE administration (Figure 4i).

### 2.5. AAE Inhibited Polyol Pathway Activity and Glycogen Accumulation in HFD/STZ Mice

**Figure 5:**
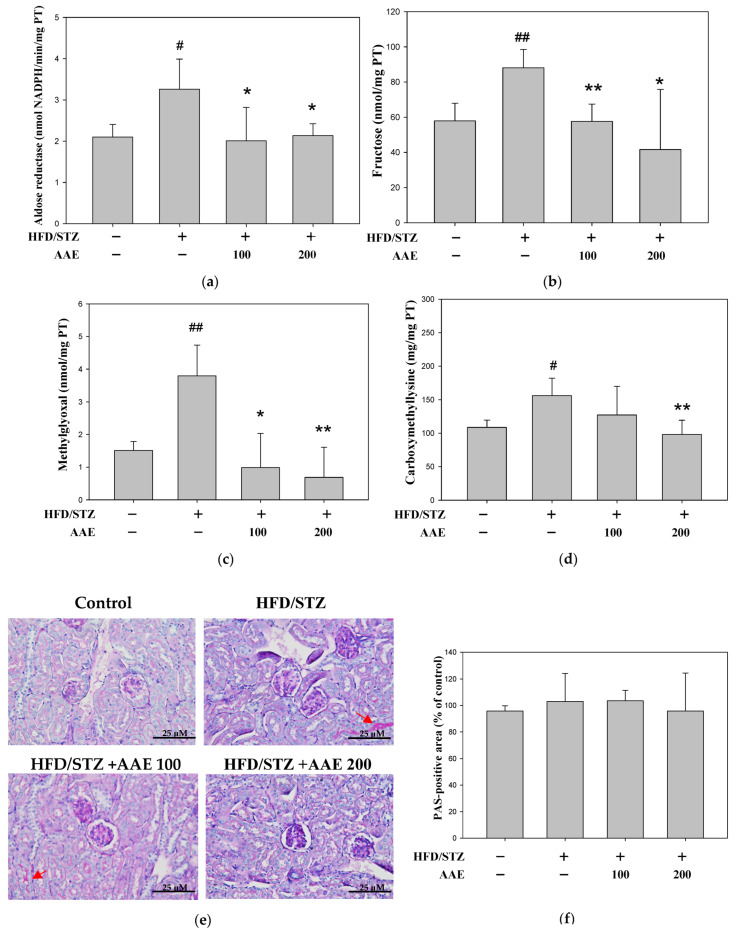
AAE inhibited polyol pathway activity and glycogen accumulation in HFD/STZ mice. The levels of aldose reductase (**a**), fructose (**b**), methylglyoxal (**c**), and carboxymethyl-lysine (**d**) were detected by a commercially available ELISA kit. (**e**) Pathological changes were evaluated by periodic acid–Schiff (PAS) staining, showing glycogen accumulation and abnormal polysaccharides (400× magnification). (**f**) PAS-positive areas in kidney tissues were quantified. Data were collected per group and are presented as the mean ± SD (*n* = 5), derived from five independent biological replicates. ^#^ *p* < 0.05, ^##^ *p* < 0.01 versus the control group; * *p* < 0.05, ** *p* < 0.01 versus the model group. AAE: *Artemisia indica* Willd. aqueous extract; HFD: high-fat diet; PAS: periodic acid–Schiff; STZ: streptozotocin.

Our results showed that AAE treatment suppressed aldose reductase (Figure 5a) and reduced renal fructose compared with HFD/STZ mice (Figure 5b). As presented in Figure 5c, AAE administration dose-dependently decreased renal methylglyoxal. Carboxymethyl-lysine (CML) was elevated in the HFD/STZ group relative to the controls; however, this increase was reversed by AAE, which also demonstrated inhibitory effects on CML (Figure 5d). Furthermore, periodic acid–Schiff (PAS) staining was performed to assess glycogen deposition. The HFD/STZ group exhibited mild increases in PAS-positive staining, reflecting subtle glycogen accumulation. AAE administration reduced this PAS reactivity (red arrows, Figure 5e). Quantitative analysis further demonstrated a decreasing trend in the PAS-positive area in the AAE-treated groups (Figure 5f).

### 2.6. AAE Enhanced Antioxidant Defense in HFD/STZ Mice

**Figure 6:**
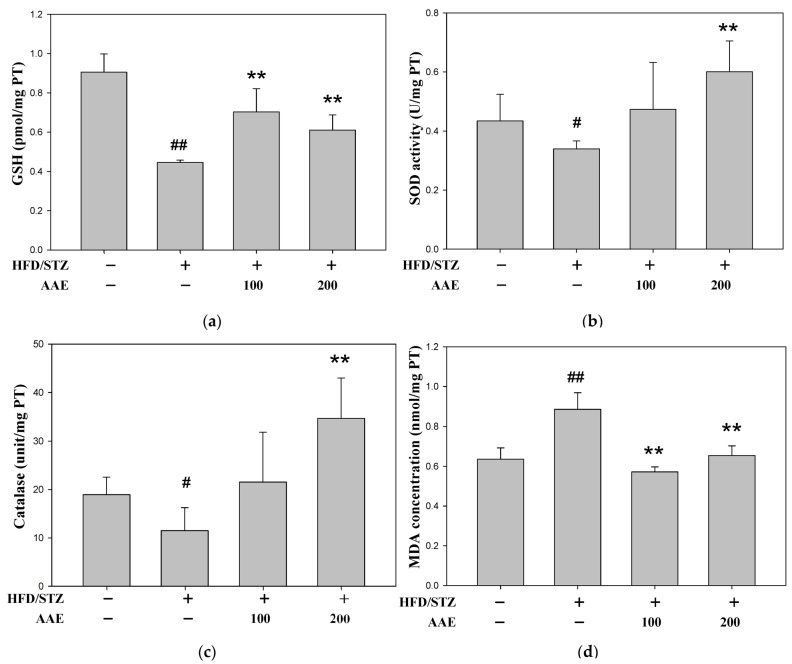
AAE enhanced antioxidant defense in HFD/STZ mice. The content of antioxidant enzymes, including glutathione (GSH) (**a**), superoxide dismutase (SOD) (**b**), and catalase (**c**), was detected by ELISA assays. (**d**) Malondialdehyde (MDA). Data were collected per group and are presented as the mean ± SD (*n* = 5), derived from five independent biological replicates. ^#^ *p* < 0.05, ^##^ *p* < 0.01 versus the control group; ** *p* < 0.01 versus the model group. AAE: *Artemisia indica* Willd. aqueous extract; HFD: high-fat diet; STZ: streptozotocin.

The activities of glutathione (GSH) (Figure 6a), superoxide dismutase (SOD) (Figure 6b), and catalase (Figure 6c) were significantly reduced in HFD/STZ-induced mice. AAE treatment restored these antioxidant enzyme activities, with a more pronounced effect at a higher dose. Furthermore, the increase in lipid peroxidation, measured by malondialdehyde (MDA), was reversed by both low- and high-dose AAE treatment (Figure 6d).

## 3. Discussion

*Artemisia* has shown potential in mitigating renal injury. Extracts such as those of *Artemisia asiatica* and *Artemisia annua* have been reported to attenuate nephrotoxicity by modulating key pathways, including MAPKs/Caspase-3-mediated apoptosis in cisplatin-induced injury and COX-2/NF-κB signaling in gentamicin-induced injury [11,12]. In our study, HPLC-ESI-MS/MS analysis identified dicaffeoylquinic acid derivatives as the major constituents of AAE (Figure 1 and Table 1). Previous studies reported that extracts rich in dicaffeoylquinic acids such as those derived significantly reduced xanthine oxidase activity and protected renal function [13]. Moreover, 4,5-dicaffeoylquinic acid (isochlorogenic acid C) possesses antimicrobial, antioxidant, and anti-inflammatory properties [14,15,16], and recent studies indicate that it can alleviate high-fat-diet-induced hyperlipidemia in the liver [15]. Research has shown that natural compounds derived from herbs and plant extracts offer advantages such as lower toxicity and fewer side effects. Given its high abundance in AAE and its previously reported bioactivities, 4,5-dicaffeoylquinic acid is likely a key contributor to the metabolic improvements observed in our study, particularly the amelioration of glucose and cholesterol dysregulation. These observations suggest a key mechanistic role for dicaffeoylquinic acid, warranting further investigation in future studies.

High-fat diets can cause metabolic dysregulation, adipose tissue dysfunction, and excessive lipid accumulation in the kidneys. Dysglycemia also contributes to oxidative stress by ROS production, which can exacerbate metabolic complications, including renal impairment. Fluctuations in blood glucose are closely associated with dyscholesterolemia, and more than half of patients present with abnormal lipid profiles [17]. Moreover, disorder of lipid metabolism can directly injure renal cells through lipotoxic mechanisms or indirectly accelerate systemic metabolic dysregulation [18,19]. In this study, mice fed a high-fat diet (HFD) combined with low-dose STZ injection exhibited hallmark features, including increased body weight (Figure 2d), elevated blood glucose (Figure 2e), and insulin resistance (Figure 2f), indicating that the HFD/STZ model effectively recapitulates the pathological characteristics of dysglycemia, insulin resistance, and metabolic abnormalities. Although blood glucose levels in the study did not reach the diabetic range, the HFD/STZ model clearly demonstrated statistically significant glucose metabolic disturbances (Figure 2). This observation is consistent with previous reports indicating that certain mouse strains exhibit partial resistance to HFD-induced metabolic disturbances, including alterations in triglyceride levels. Unlike the commonly used diabetes-prone C57BL/6J strain, BALB/c mice are classified as relatively resistant to HFD-induced obesity, hyperglycemia, and hyperlipidemia, which likely accounts for the mild metabolic phenotype observed in the present study [20]. In this study, serum levels of total cholesterol (TC), low-density lipoprotein cholesterol (LDL-c), and the LDL-c/high-density lipoprotein cholesterol (HDL-c) ratio (Figure 4a,d,f), as well as renal lipid droplet accumulation assessed by Oil Red O staining (Figure 4g,h), were markedly elevated in HFD/STZ-challenged mice. Notably, no significant difference in serum triglyceride levels was observed between the HFD/STZ and control groups (Figure 4b). These findings indicate that the experimental model recapitulated a state of dyscholesterolemia rather than classical dyslipidemia, suggesting that the suitability of this mouse strain for modeling typical dyslipidemia under HFD/STZ conditions warrants further investigation. In our study, the STZ/HFD-treated mice, when compared with the corresponding matched control group, demonstrated elevations in key glucose and lipid parameters, consistently indicating the induction of mild metabolic dysregulation. In addition, we noted that the HDL-c levels in the STZ/HFD group exhibited an unexpectedly increasing tendency (Figure 4c). To better interpret the profile, we calculated the TC/HDL-c ratio, as relying solely on total cholesterol or HDL-c may not accurately reflect cholesterol imbalance. The TC/HDL-c ratio is also widely used in clinical practice as a superior indicator of metabolic abnormalities. Our results showed that the TC/HDL-c ratio was elevated in the STZ/HFD group and was reduced following AAE treatment (Figure 4e), supporting the potential of AAE in robustly modulating lipid metabolism. Clinical evidence has extensively demonstrated that reducing lipid synthesis and serum cholesterol levels is effective for the prevention and early treatment of diabetes [21]. In line with these findings, AAE treatment markedly reduced intracellular lipid accumulation (Figure 4) and demonstrated strong therapeutic potential in mitigating induced cellular injury.

Aldose reductase, the initial and rate-limiting enzyme in the polyol pathway, reduces glucose to sorbitol using NADPH as a cofactor, which is then metabolized to fructose by sorbitol dehydrogenase [22]. Fructose, an important downstream product of the polyol pathway, serves as a key indicator of pathway activation, and its excessive accumulation contributes to cellular injury. Methylglyoxal, a highly reactive advanced glycation end-product (AGE) precursor, and carboxymethyl-lysine, a major AGE adduct, are two important indicators of glycation-associated oxidative stress. In addition, AAE inhibited the formation of methylglyoxal and carboxymethyl-lysine (Figure 5c,d). In addition, previous studies have preliminarily demonstrated that the chloroform, ethyl acetate, and n-butanol fractions of *Artemisia indica* Willd. exert hypoglycemic effects, as well as protective actions on hepatic and renal function in diabetic rats. However, the underlying molecular mechanisms remain incompletely understood [8].

Moreover, an acute oral toxicity study of AAE (methanol extract) revealed no mortality in mice, even at a dose of 2000 mg/kg bw [8]. Upregulation of key antioxidant enzymes such as superoxide dismutase (SOD), catalase, and glutathione (GSH) plays a critical role in protecting against oxidative damage and mitigating the progression of metabolic disorders. Maintaining cellular redox equilibrium is therefore regarded as an essential strategy for preserving renal function under dysglycemic conditions. Natural compounds or plant-derived extracts with strong antioxidant potential may confer broad renoprotective benefits by attenuating oxidative injury in the kidney. Phytochemical characterization identified 4,5-dicaffeoylquinic acid as the predominant constituent of AAE (Figure 1), which may be associated with the antioxidant and metabolic effects observed in this study. Administration of AAE alleviated fasting blood glucose levels and ameliorated insulin resistance in a dose-dependent manner (Figure 2e,f). AAE treatment appeared to support antioxidant defenses, as evidenced by the observed increases in SOD, catalase, and GSH activities. This was concomitant with a reduction in lipid peroxidation, as reflected by the decrease in MDA levels (Figure 6). Compared with dosages reported in previous studies, the 200 mg/kg dose used in this study is relatively modest [8]. Nutritional hormesis manifests as a biphasic dose–response relationship; whereas excessive intake may impair antioxidant defenses, optimal low-dose exposure appears to elicit adaptive responses that enhance cellular stress resistance. These findings are consistent with the concept of nutritional hormesis, whereby non-toxic levels of bioactive compounds elicit adaptive cellular responses that enhance stress resistance. While excessive exposure to certain phytochemicals may be detrimental, appropriate doses can activate endogenous protective mechanisms involved in antioxidant and anti-inflammatory regulation. Accumulating evidence indicates that hormetic natural compounds exert beneficial effects by activating antioxidant enzymes and modulating stress-responsive signaling pathways in both in vitro and in vivo models [23,24]. Previous studies have also described natural compounds, referred to as hormetic nutrients, which confer protection during metabolic disturbances through the activation of antioxidant enzymes or the suppression of NF-κB expression [25,26,27]. In this context, our results suggest that 4,5-dicaffeoylquinic acid, as a major component of AAE, may contribute to the activation of intrinsic redox defense systems at physiologically relevant doses. Although the metabolic alterations induced by HFD/STZ in BALB/c mice were relatively mild, AAE administration at 200 mg/kg consistently exhibited protective trends through coordinated modulation of oxidative stress. This adaptive stress–response framework may therefore hold relevance for the mitigation of chronic disorders associated with metabolic imbalance and impaired antioxidant capacity.

We acknowledge several limitations in the present study that warrant consideration. First, the pharmacokinetics of AAE, including absorption, metabolism, and distribution, may further influence its efficacy and therapeutic outcomes. While individual constituents often act on a limited molecular target, the whole extract has the potential to simultaneously modulate multiple pathological pathways. Notably, most preclinical studies support the superior efficacy of plant extracts or their active constituents owing to their multi-component and multi-target actions [28,29,30]. A limitation of our study is that the observations remain focused on the modulation of specific protein expression levels. Consequently, we could not definitively establish whether these alterations reflect direct regulatory effects on integrated lipid metabolic or inflammatory pathways. Consequently, further functional and mechanistic validation will be required to substantiate these preliminary findings. The unifying mechanism of hormetic-induced chemoprevention is widely recognized to involve the activation of the Nrf2 (nuclear factor erythroid 2-related factor 2) signaling pathway, which coordinates the expression of numerous cytoprotective genes [23]. While our preliminary findings demonstrate that AAE appears to modulate NF-κB expression, its precise role in activating the Nrf2-mediated antioxidant response remains to be fully elucidated. Therefore, further investigations are warranted to confirm whether the protective effects of AAE are mediated through the Nrf2/HO-1 pathway, providing a more comprehensive understanding of its hormetic potential. We also acknowledge that neutrophil gelatinase-associated lipocalin (NGAL) is a well-established biomarker of renal tubular injury and could serve as an informative indicator in future investigations. This study provides new insights into the therapeutic potential of AAE, demonstrating its capacity to modulate multiple pathways involved in renal injury. In addition, the pharmacokinetic properties and oral bioavailability of AAE have not yet been evaluated. These parameters are critical and warrant further investigation in future preclinical and clinical studies. Moving forward, the potential of AAE as a novel therapeutic agent for managing metabolic dysregulation should be further investigated in clinical studies to confirm its efficacy and safety.

## 4. Materials and Methods

### 4.1. Preparation of Plant Samples for HPLC-ESI-MS/MS Analysis of Artemisia indica Willd

*Artemisia indica* Willd., which belongs to the *Asteraceae* family, was collected in Taichung, Taiwan, and identified by the Herbarium of Taiwan biodiversity research institute, under register number 051489. To prepare the sample, 300 g of dried *Artemisia indica* Willd. leaves was macerated and then boiled (100 °C, 1500 mL) for 1 h. Then, the decoction was filtered and re-boiled, and the process was repeated three times. The filtrates were then concentrated and lyophilized under vacuum at −85 °C. The yield of *Artemisia indica* Willd. aqueous extract (AAE) was approximately 12.2% of dried materials, stored at −80 °C before experimental use. The AAE was processed using a Waters Symmetry column and a Security-Guard Ultra C18 column (Hewlett-Packard, Palo Alto, CA, USA). Separation was achieved through gradient elution with solvent A (25 mM ammonium acetate with 0.1% formic acid) and solvent B (acetonitrile with 0.1% formic acid) by HPLC-ESI-MS/MS analysis. The elution program involved an isocratic phase at 5% solvent B for the 1st minute, followed by a gradient increase from 5% to 15% B over the next 4 min, then to 95% B from 5 to 15 min, concluding with a 10 min isocratic phase at 95% B. The absorption spectra were recorded within the range of 210 to 600 nm using an in-line PDA detector (Hewlett-Packard, Palo Alto, CA, USA). A triple quadrupole mass spectrometer, operating in electrospray ionization with negative ionization mode at −3700 V, was used to identify active compounds. Sample extracts from solid-phase extraction were injected into the column using an autosampler (Hewlett-Packard, Palo Alto, CA, USA). Compound identification was achieved with authentic standards [31].

### 4.2. Animal Experiments

Six-week-old BALB/cByJNarl mice were purchased from Bio LASCO Taiwan Co., Ltd. (Taipei, Taiwan). All the animal use protocols were approved by the local ethics committee and the Institutional Animal Care and Use Committee (IACUC approval number: 1835) of Chung Shan Medical University, and the experiments complied with the relevant regulations. After acclimatization to laboratory conditions for one week, BALB/cByJNarl mice were randomly divided into four groups in a balanced manner (6 mice per group) as follows: a control group, high-fat diet (HFD) combined with streptozotocin (STZ)-induced group, HFD combined with STZ-induced +AAE 100 mg/kg bw group, and HFD combined with STZ-induced +AAE 200 mg/kg bw group. Measurement sequences were randomly adjusted, and procedures for each group were conducted at consistent time intervals. The control groups and AAE-alone group were fed a regular chow diet (#5010; LabDiet, St. Louis, MO, USA), and the others were fed a high-fat diet (DIO rodent purified diet with 60% energy from fat—blue 58Y1, Test Diet^®^, Richmond, IN, USA). After 6 weeks, the HFD mice received continuous STZ (Seelze, Germany, Cat#S0130 SIGMA), administered at a dose of 40 mg/kg body weight via intraperitoneal injection for five consecutive days [32]. After STZ induction for five days, an oral glucose tolerance test (OGTT) was performed after 12 h of food deprivation. Mice were administered an oral glucose solution at a dose of 2 g/kg. Blood samples were collected from the tail vein at 30, 60, 90, and 120 min post administration to assess blood glucose levels in each group. After STZ induction, the HFD/STZ mice were orally administered AAE (100 or 200 mg/kg bw) once daily. After 6 weeks of treatment, all mice were euthanized by CO2, and serum and urine samples were measured with an auto-analyzer. Fasting blood glucose was measured immediately after sacrifice.

### 4.3. Histological Examination

Kidney paraffin sections were prepared and stained with a hematoxylin–eosin staining kit (BioVision, Milpitas, CA, USA) and a periodic acid–Schiff staining kit (Abcam, Cambridge, MA, USA) according to the manufacturers’ instructions [33]. The mean glomerular volume for individual kidney samples was calculated using the following formula: glomerular volume = β/K[AG] 3/2. In this equation, β represents the size distribution coefficient (β = 1.38), and K is the shape coefficient (K = 1.1) for glomeruli idealized as a sphere [34]. Histological changes were observed at 400× optical magnification. ImageJ software (version 1.51k; National Institutes of Health, Bethesda, MD, USA) determined the glomerulus size and PAS-positive mesangial area. The tissue sections were stained with Oil Red O working solution (dissolved in 100% isopropanol) [35]. After 5 min, the sections were observed under a microscope (Olympus BX53 microscope, Olympus Corporation, Tokyo, Japan) and quantified with ImageJ.

### 4.4. Assessment of Biochemical Parameters

Serum samples were analyzed using a biochemical analyzer (Hitachi 7020 Chemistry Analyzer, Hitachi Co., Ltd., Tokyo, Japan) to evaluate the following biochemical markers: homeostatic model assessment of insulin resistance (HOMA-IR), fructoamine, urea nitrogen (BUN), creatinine, total cholesterol (CHOL), triglycerides (TGs), low-density lipoprotein cholesterol (LDL-c), and high-density lipoprotein cholesterol (HDL-c). In addition, urine proteins were assessed by SDS-PAGE followed by Coomassie blue staining. A normalized analysis was conducted by measuring urinary albumin (Mouse Albumin AssayMax ELISA Kit, Assaypro, Saint Charles, MO, USA, Cat# EMA3201-1) and urinary creatinine (Cayman Creatinine ELISA Kit, Item No. 502330, Michigan, USA), and subsequently, the urinary albumin-to-creatinine ratio (UACR) was calculated [36].

### 4.5. Carboxymethyl-Lysine (CML) Formation

Following the manufacturer’s instructions [37], carboxymethyl-lysine (CML) was measured in the serum using competitive ELISA kits (OxiSelect Methylglyoxal Competitive ELISA Kit and OxiSelect N-epsilon-(Carboxymethyl) Lysine Competitive ELISA Kit, purchased from CELL BIOLABS Inc. (San Diego, CA, USA)).

### 4.6. Analysis of Polyol Pathway Activation

Polyol pathway activation was assessed by measuring aldose reductase (AR) activity and renal fructose levels. AR activity was determined using a colorimetric Aldose Reductase Activity Assay Kit (Abcam, Cat. ab273276). Kidney tissues were homogenized in assay buffer, and the supernatants were loaded with NADPH and reaction mixtures. The decrease in absorbance at 340 nm was normalized to protein content. Renal fructose was quantified using a commercial fructose assay kit (Abcam, Cat. ab284537). Tissue homogenates were reacted with the enzyme, and absorbance was measured at the recommended wavelength. Fructose levels were calculated from a standard curve and normalized to tissue weight.

### 4.7. Antioxidant Assay

The activities of glutathione (GSH), superoxide dismutase (SOD), and catalase in 5–10 μg of kidney tissue homogenate were assessed using commercial assay kits. These included a glutathione (GSH) assay kit, a superoxide dismutase (SOD) assay kit, and a catalase assay kit, all from Cayman Chemical (Ann Arbor, MI, USA) [38].

### 4.8. Lipid Peroxidation Assay

Samples were collected by centrifugation; the kidney tissue was analyzed using the TBARS assay. In the TBA test reaction, MDA reacted with thiobarbituric acid (TBA) to form a pink pigment that had an absorption maximum of 532 nm. The TBARS results were expressed as MDA equivalents, which were used to calculate the total cellular protein [39].

### 4.9. Western Blot Analysis

Kidney tissue proteins were extracted using radio-immunoprecipitation assay buffer with a protease inhibitor cocktail. Protein concentrations were determined using a commercial BCA assay kit (Pierce; Thermo Fisher Scientific, Rockford, IL, USA). Samples containing 30 μg of protein were separated by 8–15% SDS-PAGE and transferred to nitrocellulose membranes (Whatman, GE Healthcare, Freiburg, Germany). The membranes were incubated overnight at 4 °C with primary antibodies including p-PKCα, PKCα, NF-κB, SREBP-1, and FAS (all from Santa Cruz, CA, USA), as well as anti-β-actin (A5441) from Sigma-Aldrich. Following incubation with anti-mouse IgG (A9044) secondary antibodies from Sigma-Aldrich (St. Louis, MO, USA) for one hour, the membranes were treated with a chemiluminescence reagent (Millipore, Burlington, MA, USA) and visualized using an ImageQuant™ LAS 4000 mini system (GE Healthcare Bio-Sciences AB, Uppsala, Sweden).

### 4.10. Statistical Analysis

All data are presented as means ± standard deviation (SD) from five independent experiments. Data normality was assessed using the Shapiro–Wilk test. Differences among groups were analyzed by one-way analysis of variance (ANOVA), followed by Tukey’s multiple comparison test as a post hoc analysis. A *p*-value of less than 0.05 was considered statistically significant.

## 5. Conclusions

**Figure 7:**
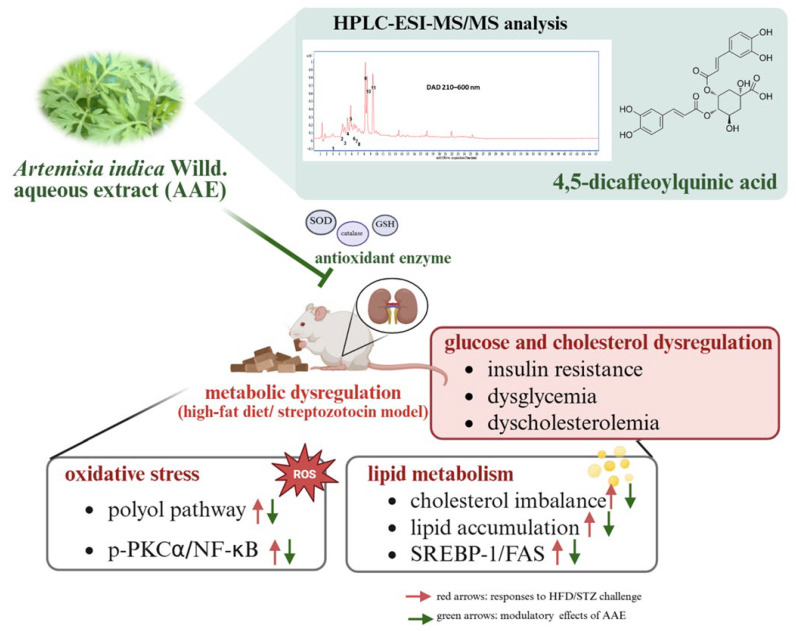
Effect of AAE in modulating metabolic dysregulation. HPLC–ESI–MS/MS identified 4,5-dicaffeoylquinic acid as the primary constituent, plausibly associated with improved glucose and cholesterol homeostasis. AAE administration was associated with attenuated oxidative stress and polyol pathway modulation, alongside ameliorated cholesterol imbalance. Collectively, these findings suggest that AAE possesses the potential to modulate multiple pathways involved in mitigating early-stage metabolic dysregulation.

This study demonstrated that the aqueous extract of *Artemisia indica* Willd. (AAE) exerts protective effects against metabolic disturbances by modulating multiple pathological pathways. HPLC–ESI–MS/MS analysis identified 4,5-dicaffeoylquinic acid as the major phytochemical constituent of AAE. AAE administration was associated with the modulation of the polyol pathway, as well as the attenuation of PKC-α and NF-κB expression. Furthermore, AAE exhibited the potential to regulate SREBP-1 and FAS expression. In addition, AAE appeared to support endogenous antioxidant enzyme activities, thereby mitigating oxidative stress. Collectively, these findings suggest that AAE possesses antioxidant and lipid-modulating properties, underscoring its potential relevance as a natural agent for the management of glucose and cholesterol dysregulation (Figure 7).

## Figures and Tables

**Table 1:** Compounds detected in aqueous extract of *Artemisia indica* Willd. by HPLC-ESI-MS/MS.

Peak No.	Rt (min)	Compound	UV-Vis λmax (nm)	MS [M − H]^−^	MS/MS	Content (mg/g Extract)
1	2.99	3-Caffeoylquinic acid	324, 294 sh	353	191, 179, 135	22.2
2	4.53	4-Caffeoylquinic acid	324, 292 sh	353	191	50.2
3	4.87	5-Caffeoylquinic acid	324, 296 sh	353	191, 173, 179, 135	18.8
4	5.32	Caffeic acid	322, 294 sh	179	135	55.8
5	5.81	Apigenin 6,8-C-pentoside-hexoside	328, 270	563	353, 383, 473, 443	80.5
6	6.39	Apigenin 6,8-di-C-pentoside	326	533	353	19.7
7	6.67	Rutin	254, 352	609	300	19.8
8	7.12	Quercetin-3-O-glucoside	330	463	300, 271	19.7
9	8.16	3,4-Dicaffeoylquinic acid	322, 296 sh, 242	515	179, 173, 191, 135	179.3
10	8.44	3,5-Dicaffeoylquinic acid	326, 298 sh, 240	515	191, 179, 135, 173	137.4
11	9.37	4,5-Dicaffeoylquinic acid	326, 298 sh, 242	515	173, 179, 191, 135	212.6

Rt: retention time.

## Data Availability

The original contributions presented in this study are included in the article. Further inquiries can be directed to the corresponding author.

## Abbreviations

The following abbreviations are used in this manuscript:

AAE	*Artemisia indica* Willd. aqueous extract
AGE	Advanced glycation end-product
BUN	Blood urea nitrogen
CML	Carboxymethyl-lysine
ESRD	End-stage renal disease
FAS	Fatty acid synthase
GSH	Glutathione
HDL-c	High-density lipoprotein cholesterol
HFD	High-fat diet
HOMA-IR	Homeostatic model assessment of insulin resistance
i.p.	Intraperitoneal injection
LDL-c	Low-density lipoprotein cholesterol
MDA	Malondialdehyde
NF-κB	Nuclear factor kappa B
NGAL	Neutrophil gelatinase-associated lipocalin
Nrf2	Nuclear factor erythroid 2-related factor 2
OGTT	Oral glucose tolerance test
PAS	Periodic acid–Schiff
PKC	Protein kinase C
ROS	Reactive oxygen species
SREBPs	Sterol regulatory element-binding proteins
STZ	Streptozotocin
TC	Total cholesterol
TGs	Total triglycerides
UACR	Urine albumin-to-creatinine ratio
